# Identification of Plasma Lipid Biomarkers for Prostate Cancer by Lipidomics and Bioinformatics

**DOI:** 10.1371/journal.pone.0048889

**Published:** 2012-11-12

**Authors:** Xinchun Zhou, Jinghe Mao, Junmei Ai, Youping Deng, Mary R. Roth, Charles Pound, Jeffrey Henegar, Ruth Welti, Steven A. Bigler

**Affiliations:** 1 Department of Pathology, University of Mississippi Medical Center, Jackson, Mississippi, United States of America; 2 Department of Biology, Tougaloo College, Tougaloo, Mississippi, United States of America; 3 Department of Computer Sciences, Georgia State University, Atlanta, Georgia, United States of America; 4 Department of Medicine, Rush University Medical Center, Chicago, Illinois, United States of America; 5 Kansas Lipidomics Research Center, Division of Biology, Kansas State University, Manhattan, Kansas, United States of America; 6 Department of Surgery, University of Mississippi Medical Center, Jackson, Mississippi, United States of America; 7 Department of Pathology, Mississippi Baptist Medical Center, Jackson, Mississippi, United States of America; Ottawa Hospital Research Institute, Canada

## Abstract

**Background:**

Lipids have critical functions in cellular energy storage, structure and signaling. Many individual lipid molecules have been associated with the evolution of prostate cancer; however, none of them has been approved to be used as a biomarker. The aim of this study is to identify lipid molecules from hundreds plasma apparent lipid species as biomarkers for diagnosis of prostate cancer.

**Methodology/Principal Findings:**

Using lipidomics, lipid profiling of 390 individual apparent lipid species was performed on 141 plasma samples from 105 patients with prostate cancer and 36 male controls. High throughput data generated from lipidomics were analyzed using bioinformatic and statistical methods. From 390 apparent lipid species, 35 species were demonstrated to have potential in differentiation of prostate cancer. Within the 35 species, 12 were identified as individual plasma lipid biomarkers for diagnosis of prostate cancer with a sensitivity above 80%, specificity above 50% and accuracy above 80%. Using top 15 of 35 potential biomarkers together increased predictive power dramatically in diagnosis of prostate cancer with a sensitivity of 93.6%, specificity of 90.1% and accuracy of 97.3%. Principal component analysis (PCA) and hierarchical clustering analysis (HCA) demonstrated that patient and control populations were visually separated by identified lipid biomarkers. RandomForest and 10-fold cross validation analyses demonstrated that the identified lipid biomarkers were able to predict unknown populations accurately, and this was not influenced by patient's age and race. Three out of 13 lipid classes, phosphatidylethanolamine (PE), ether-linked phosphatidylethanolamine (ePE) and ether-linked phosphatidylcholine (ePC) could be considered as biomarkers in diagnosis of prostate cancer.

**Conclusions/Significance:**

Using lipidomics and bioinformatic and statistical methods, we have identified a few out of hundreds plasma apparent lipid molecular species as biomarkers for diagnosis of prostate cancer with a high sensitivity, specificity and accuracy.

## Introduction

Biomarkers play pivotal roles in the care of patients with cancers. However, currently used biomarkers for prostate cancer are sub-optimal. For example, prostate specific antigen (PSA), the most widely used biomarker, is controversial with regard to its specificity and sensitivity in various populations [1]. There are also concerns regarding possible over-diagnosis of prostate cancer by PSA in patients with limited potential for disease progression [2]–[4]. Several new biomarkers are being studied [5]–[7]; however, none of these has proven to be useful for clinical testing.

Lipids comprise diverse classes of molecules with critical functions in cellular energy storage, structure, and signaling. Previous studies have demonstrated that the risk of prostate cancer is increased with elevations in particular plasma fatty acids, such as myristic acid, α-linolenic acid, and eicosapentaenoic acids [8]–[9]. Many individual polar lipid [10]–[17] and cholesterol [18]–[21] species have been associated with the evolution of prostate cancer. However, due to limitations in technology, only a few apparent lipid species or lipid classes were analyzed in each of these studies, and no attempt has been made to discover lipid molecules as biomarkers for prostate cancer by large scale lipid profiling. Large scale lipid profiling was not done until the introduction of mass-spectrometry-based lipidomics strategies a decade ago [22]. Recently, reference values for 500 plasma lipid species were obtained from a lipidomics analysis of the pooled and blended plasma from 100 healthy people [23]. Lipidomics has been demonstrated to be a useful tool in the study of mechanisms and biomarkers in many diseases such as obesity [24]–[25], atherosclerosis [26]–[28], hypertension [29], diabetes [30], cystic fibrosis [31] and other cancers [32]–[34]. As well, using shotgun lipidomics, a few lipid species from 70 phospholipids species in urine were identified as potential markers for prostate cancer [35]. However, large scale plasma lipid profiling has not been performed on blood and prostatic samples from patients with prostate cancer.

As described herein, we performed a global lipid profiling, which included measurement of 340 phospholipid and 50 cholesteryl ester (CE) apparent lipid molecular species, on 141 plasma samples from 105 patients with prostate cancer and 36 male controls. By analysis with bioinformatic and statistical methods, a few plasma lipid species have been selected as biomarkers. The initial study demonstrates that these biomarkers have a high sensitivity, specificity and accuracy in diagnosis of prostate cancer.

## Patients and Methods

### Objectives

We hypothesize that prostate cancer tissues have distinct lipid profiles to meet special needs for tumor survival and progression. Distinctive lipid profiles will influence systemic lipid homeostasis and be reflected in body fluids including plasma. Therefore, detection of plasma apparent lipid molecular species will reflect the existence and progression of prostate cancer. By comparing plasma concentrations of hundreds of apparent lipid species among populations with and without prostate cancer, a few apparent lipid species that are the most representative of cancer status will be identified as plasma lipid biomarkers in diagnosis of prostate cancer.

### Patients and Sample Collection

One hundred and five (105) plasma samples from 105 patients with prostate cancer were obtained from the Cooperative Human Tissue Network (CHTN), where plasma samples were pre-collected from different clinics during the period from 2004–2007. Before collection of plasma samples, patients had given consent and had not undergone therapeutic interventions. Diagnosis of prostate cancer for each patient was confirmed by subsequent prostate biopsy or prostatectomy. Limited information for each patient, including the patient's age, race and pathological diagnosis, was provided. Thirty six (36) plasma samples from male controls were obtained from a collaborating author, who pre-collected the plasma from 36 male patients at a community clinic, where patients had their wellness checks or sought for medical help for other diseases during the period 2006–2008. Criteria for selection of controls were no history of prostate cancer, denial of clinical manifestations of prostate cancer, and a low level of serum PSA. The same protocol for collection of plasma was used in collection of plasma samples from patients and controls: from each subject, 10 ml whole blood was collected into a vacutainer tube containing potassium-EDTA as anticoagulant. The plasma was promptly separated (no more than 4 h after collection of whole blood) and stored at −80°C immediately. All plasma samples were transported on dry ice to the Kansas Lipidomics Research Center (KLRC) for lipid analysis.

### ESI-MS/MS Lipid Profiling

An automated electrospray ionization-tandem mass spectrometry approach was used. In this approach, plasma lipid species are identified at level of head group plus total acyl carbons: total double bonds. The detected intensities, each defined by an intact ion mass/charge (m/z) and a characteristic fragment m/z, are herein described as “apparent lipid molecular species”. Data acquisition and analysis were carried out as described previously [36]–[37] with modifications. Briefly, an aliquot of 3 µl of plasma was used. Precise amounts of internal standards, obtained and quantified as previously described [38], were added in the following quantities (with some small variation in amounts in different batches of internal standards): 0.60 nmol PC(12∶0/12∶0), 0.60 nmol PC(24∶1/24∶1), 0.60 nmol LPC(13∶0), 0.60 nmol LPC(19∶0), 0.30 nmol PE(12∶0/12∶0), 0.30 nmol PE(23∶0/23∶0), 0.30 nmol LPE(14∶0), 0.30 nmol LPE(18∶0), 0.30 nmol LPG(14∶0), 0.30 nmol LPG(18∶0), 0.30 nmol PA(14∶0/14∶0), 0.30 nmol PA (phytanoyl/phytanoyl), i.e. PA(20∶0/20∶0), 0.20 nmol PS(14∶0/14∶0), 0.20 nmol PS(phytanoyl/phytanoyl), i.e. PS(20∶0/20∶0), 0.23 nmol PI (16∶0/18∶0), 2.5 nmol CE(13∶0) and 2.5 nmol CE(23∶0). The sample and internal standard mixture were combined with solvents, such that the ratio of chloroform/methanol/300 mM ammonium acetate in water was 300/665/35, and the final volume was 1.2 ml. This mixture, in autosampler vials, was centrifuged for 15 min to pellet particulates before presenting the lipid/solvent mixture to the autosampler. These unfractionated lipid extracts were introduced by continuous infusion into the ESI source on a triple quadrupole MS/MS (API 4000, Applied Biosystems, Foster City, CA), using an autosampler (LC Mini PAL, CTC Analytics AG, Zwingen, Switzerland) fitted with the required injection loop for the acquisition time and presented to the ESI needle at 30 µl/min.

Sequential precursor and neutral loss scans of the extracts produced a series of spectra with each spectrum revealing a set of lipid species containing a common head group fragment. Lipid species were detected with the following scans: PC, SM, and lysoPC, [M+H]^+^ ions in positive ion mode with Precursor of 184.1 (Pre 184.1); PE and lysoPE, [M+H]^+^ ions in positive ion mode with Neutral Loss of 141.0 (NL 141.0); PI, [M+NH_4_]^+^ in positive ion mode with NL 277.0; PS, [M+H]^+^ in positive ion mode with NL 185.0; PA, [M+NH_4_]^+^ in positive ion mode with NL 115.0; CE, [M+NH_4_]^+^ in positive ion mode with Pre 369.3. SM was determined from the same mass spectrum as PC (precursors of m/z 184 in positive mode) [39]–[40] and by comparison with PC internal standards using a molar response factor for SM (in comparison with PC) determined experimentally to be 0.39. Acyl, alk(en)yl (“ether-linked”) ePCs and ePEs were determined in relation to the same diacyl standards as other PC and PE species, and no response factors were applied. The collision gas pressure was set at 2 (arbitrary units). The collision energies, with nitrogen in the collision cell, were +28 V for PE, +40 V for PC (and SM), +25 V for PI, PS and PA, and +30 V for CE. Declustering potentials were +100 V for all lipids except CE, for which the declustering potential was +225 V. Entrance potentials were +15 V for PE, +14 V for PC (and SM), PI, PA, and PS, and +10 V for CE. Exit potentials were +11 V for PE, +14 V for PC (and SM), PI, PA, PS, and +10 V for CE. The mass analyzers were adjusted to a resolution of 0.7 u full width at half height. For each spectrum, 9 to 150 continuum scans were averaged in multiple channel analyzer (MCA) mode. The source temperature (heated nebulizer) was 100°C, the interface heater was on, +5.5 kV or −4.5 kV were applied to the electrospray capillary, the curtain gas was set at 20 (arbitrary units), and the two ion source gases were set at 45 (arbitrary units).

The background of each spectrum was subtracted, the data were smoothed, and peak areas integrated using a custom script and Applied Biosystems Analyst software. The data were isotopically deconvoluted, and the lipids in each class were quantified in comparison to the internal standards of that class, because various molecular species within the same class ionize similarly [22]. The first and typically every 11^th^ set of mass spectra were acquired on the internal standard mixture only. Peaks corresponding to the target lipids in these spectra were identified and molar amounts calculated in comparison to the internal standards on the same lipid class. To correct for chemical or instrumental noise in the samples, the molar amount of each lipid metabolite detected in the “internal standards only” spectra was subtracted from the molar amount of each metabolite calculated in each set of sample spectra. The data from each “internal standards only” set of spectra was used to correct the data from the following 10 samples. Finally, the data were corrected for the fraction of the sample analyzed and normalized to the sample volume to produce data in the unit of nmol/µl.

### Strategies used in Selection of Lipid Biomarkers

Two strategies were used in selecting individual apparent lipid species biomarkers from hundreds of detected species. The first strategy was filtration. To narrow the number of potential candidates from 390 apparent lipid species, the species that cannot be clinically used in diagnosis of prostate cancer were filtered, due to too low concentration to detect, insignificant difference between patient and control groups, or too closed levels of plasma concentrations in two groups (although the difference may be statistically significant) to interpret. Criteria for retention were: 1) difference in mean plasma lipid concentration is highly significant (p<0.01) between patient and control groups; 2) changes in mean plasma lipid concentration is >2-fold (up or down); and 3) mean plasma lipid concentration is >10 nmol/µl. Apparent lipid species that fulfilled all three criteria were selected as potential candidates of plasma lipid biomarkers. The second strategy provided additional differentiation of cancer and control samples, in order to demonstrate that the selected candidates are not only clinically useful and applicable, but also they are highly sensitive, specific and accurate in differentiation of prostate cancer from controls. After analysis with bioinformatics methods, any apparent lipid species of selected potential candidates will be selected as individual plasma lipid biomarker in diagnosis of prostate cancer, if it met these criteria: 1) sensitivity above 80%; 2) specificity above 50%; 3) all of Precision, Recall, F-measurement and Area under (ROC) curve above 80%.

### Software and Programs Used in Statistical and Bioinformatics Analysis

The T-Test in SPSS18 software was used to compare mean plasma concentrations of 390 apparent lipid species between control and patient groups. The significant p value was set at 0.01 in filtration procedures. The T-Test was also used in comparison of mean ages between control and patient groups. The significant p value was set at 0.05.

GenSpring11, Gim2 and Windows Paint software and programs were used to perform and graph charts of Principal Component Analysis (PCA) and Hierarchical Clustering Analysis (HCA).

Weka 3.73 version software was used in bioinformatics analysis: Simple logistics classification algorithm and InfoGain, a supervised attribute ranking method were used to rank individual apparent lipid species and lipid class according to their predictive powers in differentiation of patients with prostate cancer from the controls; RandomForest classification algorithm and 10-fold cross validation were used to estimate the performance of a predictive model. For “unknown prediction”, models were established in a training set, which contained populations with “known features”, such as white patient. All subjects within the same “known feature”, such as white patient, were randomly grouped (10 iterations in this study). The predictive powers were repeatedly cross validated among 10 iterations. The program determined average predictive power, which indicated if a satisfactory model was established in the training set. Then the satisfactory model with each “known feature” was used to predict (to validate) predictive power in subjects with each mirrored “unknown feature”, such as black patient, in the test set. Higher predictive power in the training set indicates smaller variances among 10 randomly grouped iterations with “known feature”. Higher predictive powers in the test set suggest a smaller variance between the paired “known” and “unknown” populations.

Chi-Square test in SPSS 18 software was used to compare the distribution of controls and patients between the first half (top portion) and the second half (bottom portion) with higher plasma lipid concentrations in Figure S1. Chi-Square test was also used to compare the ratios of Black to White, biopsy to prostatectomy, high and low grade of prostate cancer between the first and second halves. The significant p value was set at 0.05 for all results from Chi-Square tests.

### Ethics

This study was approved by the Institutional Review Board at the University of Mississippi Medical Center as an exempt investigation, in which obtaining consent from participants was not required, because all of obtained specimens used in this study were pre-collected by other organizations. The protocols for sample collection had been approved by their Institutional Review Boards. Patients had given their written consent before donation of plasma samples. In this study, no identifiable information, such as patient's name, birth date or contact information was known or used.

## Results

### Demographics of Subjects

The entire study included 141 subjects including 105 patients with prostate cancer and 36 male controls. Among the patients, 61 (58.1%) were Caucasian (“white”), 42 (40%) were African American (“black”); and 2 were of unknown ethnic origins. Among the 36 controls, 24 (66.7%) were Caucasian and 12 (33.3%) were African American. There was no difference in racial ratio between patient and control groups (p = 0.55), although there were significantly more Caucasians than African Americans in the entire study (85 Caucasians, and 54 African Americans, p = 0.0022). The average age at time of sample collection was 63.6±8.5 for patients and 57.5±15.3 for controls (p = 0.0032). Information on Gleason grade was available for 100 out of 105 patients: 2 patients had Gleason score of 5, 55 had Gleason score of 6, 35 had Gleason score of 7, and 8 had Gleason score above 7. Thus, 90% of patients had Gleason score of 6 or 7. Information on other clinical conditions that might influence lipid metabolism such as hyperlipidemia, diabetes, other malignancies and medications was not available for patients. Clinical information for 36 male controls was as following: 10 patients had their wellness check and denied significantly clinical manifestations; 14 had hypertension; 6 had osteoarthritis; and 1 to 2 patients had a history of one or more of following: hyperlipidemia, back pain, obesity, diabetes, Gout, bipolar disorder, seizure, or gastroesophageal reflux disease. None had a history of cancer including prostate cancer. The mean serum PSA level for controls was 0.85 ng/ml.

### Lipid Profiling of 390 Apparent Lipid Species

Plasma lipid profiles including 390 individual apparent lipid species from 13 classes of phospholipids and cholesteryl-esters (CE) were determined by lipidomics for 141 plasma samples (105 from patients and 36 from controls). The most significant difference in mean plasma concentration between patient and control groups was found in lysophosphatidylcholine (LPC) with the fatty acyl chain 22∶6 [LPC(22∶6)] (p = 1.75×10^−20^). The highest mean plasma concentration of a cholesteryl-ester species was CE(18∶2) (11.5±4.36 nmol/µl in patients, 10.99±4.8 nmol/µl in controls, p = 0.144). The highest mean plasma concentration of a polar lipid species was PC(34∶2) (0.94±0.41 nmol/µl in patients, 0.58±0.17 nmol/µl in controls, p = 0.00115). The significant fold change in mean plasma concentration of these apparent lipid species between patient and control groups ranged from positive 22.7-fold for dihydrosphingomyelin (DSM) with 16∶0 [(DSM(16∶0)] (p = 3.85×10^−19^) to negative 20.8-fold for PC(30∶1) (p = 9.58×10^−04^). The complete list of mean plasma concentrations in patient and control groups, p values and fold changes between patient and control groups for each of 390 species is provided in Table S1.

### Identification of Individual Apparent Lipid Species as Biomarker

According to the criteria described in the Patients and Methods section, 335 out of 390 species were removed by the first strategy, filtration. Only 35 apparent lipid species were selected as potential candidates of lipid biomarkers for diagnosis of prostate cancer (Table 1). For these 35 selected apparent lipid species, the difference in mean plasma concentration between patient and control groups was highly significant (p<0.01), the change in mean plasma concentration (increase or decrease) between patient and control groups was ≥2 fold, and the mean plasma concentration was ≥10 pmol/µl (15 times the detection cut-off value in either patients, controls, or both). The second strategy provided additional differentiation of cancer and control samples by demonstrating whether each of 35 candidates had enough predictive power in diagnosis of prostate cancer. Using bioinformatic methods, the 35 candidates were ranked as top 1 through top 35 according to their InfoGain values. Only 12 out of 35 potential candidates were identified as individual plasma lipid biomarkers, because each of these 12 identified apparent lipid species fulfilled all of criteria: sensitivity (true positive) above 80%, specificity (1-true false positive) above 50% and all of Precision, Recall, F-measurement and Area under (ROC) curve above 80% in differentiation of patients with prostate cancer from controls (as bolded and italic in Table 1).

**Table 1:** Top 35 individual plasma apparent lipid species as candidate biomarkers for prostate cancer (Concentration: pmol/µl).

Lipid		Patients (105)		Control (36)		P/C	p	Predictive Values (%)					
Species*	Rank	Mean	SD	Mean	SD	Fold	value	Sens.	Spec.	Prec.	Recall	F-meas.	AUC
***LPC(18∶1)***	***1***	***43.4***	***22.8***	***15.8***	***6.2***	***2.7***	***2.63E-09***	***80.4***	***60.6***	***79.5***	***80.4***	***79.9***	***87.4***
***LPC(20∶4)***	***2***	***14.6***	***9.4***	***4.6***	***2.0***	***3.2***	***1.71E-15***	***88.7***	***79.7***	***88.5***	***88.7***	***88.5***	***93.3***
***PC(40∶7)***	***3***	***16.9***	***10.0***	***7.6***	***3.0***	***2.2***	***4.37E-11***	***86.5***	***68.0***	***86.3***	***86.5***	***85.6***	***89.8***
***LPC(18∶0)***	***4***	***74.6***	***39.7***	***30.3***	***10.7***	***2.5***	***3.00E-08***	***80.9***	***56.9***	***79.6***	***80.9***	***79.3***	***87.9***
LPC(16∶0)	5	240.3	121.4	85.0	38.8	2.8	2.03E-06	70.3	40.8	68.1	70.3	69.0	64.1
***ePC(38∶4)***	***6***	***27.7***	***14.0***	***13.0***	***5.0***	***2.1***	***3.01E-08***	***82.3***	***66.5***	***81.6***	***82.3***	***81.8***	***87.8***
***PC(38∶4)***	***7***	***380.9***	***200.1***	***181.5***	***72.4***	***2.1***	***3.65E-05***	***80.9***	***55.1***	***79.7***	***80.9***	***79.0***	***85.4***
***PC(38∶5)***	***8***	***136.0***	***68.1***	***61.0***	***23.3***	***2.2***	***8.50E-06***	***81.6***	***60.8***	***80.5***	***81.6***	***80.5***	***84.3***
SM(18∶1)	9	67.9	45.2	22.8	14.5	3.0	1.05E-09	81.2	44.2	81.3	81.2	77.3	77.8
***SM(16∶1)***	***10***	***76.6***	***45.0***	***30.3***	***16.8***	***2.5***	***6.71E-09***	***81.2***	***50.4***	***79.8***	***81.2***	***78.8***	***81.0***
DSM(16∶0)	11	12.2	12.0	0.5	0.8	22.7	3.85E-19	74.5	25.5	55.5	74.5	63.6	50.0
***SM(16∶0)***	***12***	***494.9***	***276.2***	***200.9***	***97.3***	***2.5***	***1.20E-06***	***80.1***	***51.2***	***79.0***	***80.1***	***77.6***	***83.4***
ePC(36∶1)	13	13.3	8.9	5.5	2.0	2.4	6.30E-10	76.6	48.2	74.2	76.6	74.3	83.5
***SM(18∶0)***	***14***	***102.6***	***62.1***	***36.8***	***26.1***	***2.8***	***1.72E-06***	***80.9***	***56.9***	***79.6***	***80.9***	***79.3***	***80.3***
***ePC(36∶2)***	***15***	***25.4***	***12.3***	***12.6***	***4.9***	***2.0***	***1.72E-07***	***87.1***	***78.6***	***87.1***	***87.1***	***87.1***	***91.8***
C19∶2 CE	16	28.0	32.3	3.1	7.8	9.0	9.94E-14	73.0	54.3	72.6	73.0	72.8	74.4
ePC(38∶1)	17	10.3	6.7	4.2	1.8	2.5	4.14E-11	73.8	30.8	67.1	73.8	66.5	81.9
***LPC(18∶2)***	***18***	***61.7***	***35.2***	***28.0***	***13.5***	***2.2***	***1.26E-06***	***80.4***	***52.3***	***78.7***	***80.4***	***78.6***	***87.9***
PC(34∶1)	19	343.5	193.7	164.0	71.4	2.1	1.84E-04	73.8	39.9	69.7	73.8	70.1	82.5
SM(24∶0)	20	89.3	53.8	31.8	25.1	2.8	3.78E-06	78.0	52.3	76.1	78.0	76.2	82.7
PE(36∶2)	21	18.4	14.0	7.8	5.2	2.4	3.93E-09	78.7	47.1	77.1	78.7	75.6	82.8
C19∶3 CE	22	37.9	62.2	3.7	7.4	10.3	8.31E-11	74.5	25.5	55.5	74.5	63.6	50.0
C20∶1 CE	23	22.1	25.8	7.6	12.1	2.9	3.02E-07	74.5	25.5	55.5	74.5	63.6	50.0
C20∶0 CE	24	56.8	104.6	14.5	47.4	3.9	6.50E-07	74.5	25.5	55.5	74.5	63.6	50.0
PC(36∶1)	25	64.4	43.3	31.4	23.6	2.1	8.42E-05	75.2	33.1	71.3	75.2	68.3	67.3
C18∶0 CE	26	42.5	38.8	17.5	26.5	2.4	6.45E-04	67.4	23.1	54.0	67.4	60.0	41.9
PC(30∶1)	27	0.7	5.0	14.3	18.7	−20.9	9.58E-04	80.1	45.7	80.7	80.1	76.2	61.9
SM(22∶0)	28	16.9	44.0	63.0	78.6	−3.7	5.51E-04	77.3	46.6	74.9	77.3	74.4	63.0
C21∶3 CE	29	28.6	39.6	9.7	16.6	3.0	3.53E-06	74.5	25.5	55.5	74.5	63.6	50.0
C19∶1 CE	30	207.9	232.1	72.4	120.3	2.9	1.60E-05	74.5	25.5	55.5	74.5	63.6	51.0
C17∶0 CE	31	23.9	29.3	7.2	10.4	3.3	4.19E-05	74.5	25.5	55.5	74.5	63.6	50.0
C17∶1 CE	32	16.9	17.3	6.5	10.6	2.6	1.17E-04	74.5	25.5	55.5	74.5	63.6	50.0
C14∶0 CE	33	43.5	45.4	20.6	31.8	2.1	5.16E-03	74.5	25.5	55.5	74.5	63.6	50.0
C19∶0 CE	34	73.3	99.2	24.9	38.9	2.9	1.80E-04	74.5	25.5	55.5	74.5	63.6	50.0
C22∶5 CE	35	30.4	40.4	14.0	17.1	2.2	2.15E-03	74.5	25.5	55.5	74.5	63.6	50.0

* Apparent lipid species identities are based on the mass/charge ratio of the intact lipid ion and one characteristic fragment. Sens. = Sensitivity, Spec. = Specificity, Prec. = Precision, F-Meas. = F-measure.

### Identification of Lipid Classes as Biomarkers

The detected 390 individual plasma apparent lipid species belonged to 12 classes of phospholipids and one group of cholesteryl esters. The concentration for each lipid class was calculated by adding all of measured individual species in that class. As shown in Table 2, all lipid classes had increased plasma concentrations in patients as compared to controls except phosphatidic acid (PA), which had a significantly decreased plasma concentration in patients. LPC was the only lipid class, within which every detected individual apparent lipid species had a significantly elevated plasma concentration in patient group as compared to control group (details not shown). In the rest of the lipid classes, some apparent lipid species were up, while others were down in their plasma concentration in patients vs. controls. The differences in plasma lipid concentrations between patients and controls were statistically significant in the majority of lipid classes except lysophosphatidylethanolamine (LPE) and phosphatidylserine (PS). However, when the same criteria used in selection of biomarkers from individual apparent lipid species were applied to the lipid classes, only the lipid classes PE, ePE and ePC could be considered to be biomarkers in diagnosis of prostate cancer. Using lipid classes as biomarkers for diagnosis of prostate cancer is not an ideal choice, because potentially useful information is lost by arbitrarily combining measured values (based on lipid class assignment) for individual apparent lipid species.

**Table 2:** Lipid classes in differentiation of prostate cancer (Concentration: nmol/µl).

Lipid		Patients (105)		Controls (36)		P/C	p	Predictive values (%)					
Class	Rank	Mean	SEM	Mean	SEM	fold	value	Sens.	Spec.	Prec.	Recall	F-meas.	AUC
**LPC**	1	0.45	0.02	0.17	0.01	2.7	1.08E-11	74.5	25.5	55.5	74.5	63.6	49.3
**PE**	2	0.11	0.01	0.04	0.00	2.5	1.09E-08	83.7	59.7	83.3	83.7	82.1	85.3
**ePE**	3	0.02	0.00	0.01	0.00	2.5	1.93E-07	80.9	53.3	79.9	80.9	78.6	82.7
**SM**	4	1.24	0.07	0.57	0.03	2.2	5.73E-08	79.4	58.3	78.1	79.4	78.4	86.4
**ePC**	5	0.32	0.01	0.16	0.01	2.0	6.87E-10	81.6	59.0	80.5	81.6	80.2	89.1
**LPE**	6	0.01	0.00	0.01	0.00	1.2	4.87E-01	74.5	25.5	55.5	74.5	63.6	49.3
**PC**	7	3.69	0.15	1.94	0.10	1.9	4.23E-10	80.1	58.5	78.9	80.1	79.0	88.9
**PA**	8	0.00	0.00	0.00	0.00	−1.5	1.01E-02	75.2	29.4	73.1	75.2	66.4	53.9
**PE-Cer**	9	0.00	0.00	0.00	0.00	3.3	2.21E-06	75.9	46.1	73.1	75.9	73.3	83.0
**CE**	10	18.36	0.63	13.67	1.15	1.3	3.16E-04	76.6	35.4	75.5	76.6	70.1	61.7
**PI**	11	0.15	0.01	0.09	0.01	1.6	2.88E-05	76.6	40.9	73.9	76.6	72.3	72.2
**ePS**	12	0.00	0.00	0.00	0.00	2.3	3.40E-02	74.5	25.5	55.5	74.5	63.6	50.0
**PS**	13	0.00	0.00	0.00	0.00	1.2	6.34E-01	74.5	25.5	55.5	74.5	63.6	50.0

***Sens. = Sensitivity, Spec. = Specificity, Prec. = Precision, F-Meas. = F-measure.***

### Effect of Grouping Multiple Biomarkers on Diagnosis of Prostate Cancer

**Figure 1:**
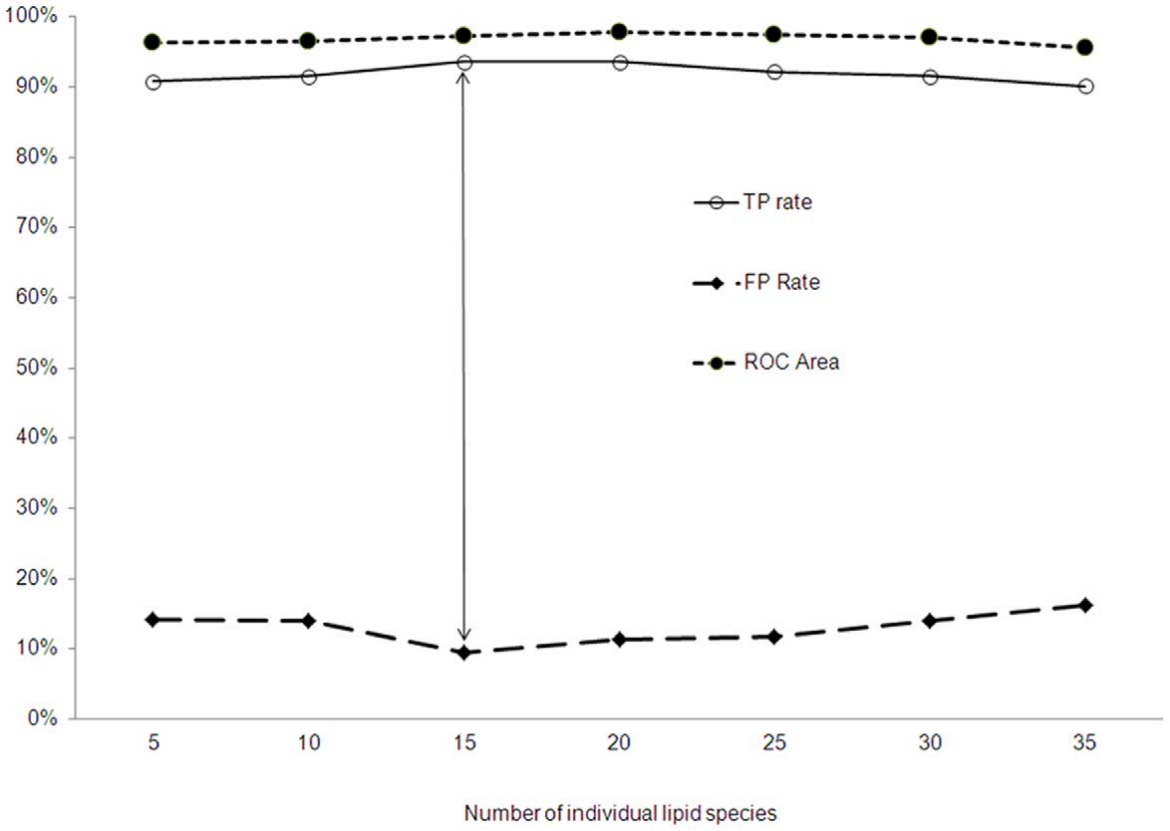
Effect of multiple individual lipid species in diagnosis of prostate cancer. The points indicated by the two head arrows are the predictive powers of top 15 plasma apparent lipid species when they are used together in diagnosis of prostate cancer. Using top 15 plasma apparent lipid species has the highest sensitivity (93.6%), the highest specificity (90.1%), and higher accuracy (ROC Area, 97.3%) in the diagnosis of prostate cancer as compared with using any other combination of different numbers.

To demonstrate if using more lipid biomarkers together within 35 candidates is able to increase the predictive power for diagnosis of prostate cancer, seven groups of apparent lipid species with different numbers of candidates were manually grouped: top 5 (top 1 through 5, 5 candidates together), 10 (top 1 through 10, 10 candidates together, etc.), 15, 20, 25, 30 and 35 (all of 35 candidates together). The results show that in any manually assembled group with more lipid markers together had higher sensitivity, specificity and accuracy in diagnosis of prostate cancer as compared to any of individual lipid biomarkers. Among these manually assembled groups, the group of 15 (top 1 through top15, 15 biomarkers together) was the best combination with the strongest predictive powers in diagnosis of prostate cancer: it had the highest sensitivity (93.6%), the highest specificity (90.1%), higher accuracy (97.3%) as shown in Figure 1, and the highest Precision, Recall and F-Measure (93.7%, 93.6% and 93.6%, respectively, data not shown in Figure 1).

To potentially reduce the number of biomarkers to facilitate practical incorporation into the workflow of clinical laboratories and to make the results most amenable to interpretation by clinicians, bioinformatics analyses were performed on the various combinations from two lipid biomarkers together (top 2) through 15 lipid biomarkers together (top 15) as shown in Table 3. As an example, if top three lipid biomarkers, LPC(18∶1), LPC(20∶4) and PC(40∶7) (Top 3) were used together, the combination would provide a sensitivity of 91.5%, specificity of 84.3% and accuracy (ROC Area) of 95.9% in differentiating patients with prostate cancer from male controls. Grouping lipid classes, as opposed to individual species, also increased sensitivity, specificity, and accuracy in diagnosis of prostate cancer as compared to any single lipid class. For example, the top 2 classes of lipids (class LPC and PE) together had a sensitivity of 88.7%, a specificity of 81.5% and an accuracy of 94.4% (data not shown). However, the predictive powers from grouping more lipid classes were lower than from grouping multiple individual plasma apparent lipid species.

**Table 3:** Comparison of predictive powers among groups with different numbers of identified plasma lipid biomarkers.

Numbers of	Predictive values (%)					
Biomarkers	Sensitivity	Specificity	Precision	Recall	F-measure	ROC Area
**Top2**	87.9	83.1	88.3	87.9	88.1	94.0
**Top3**	91.5	84.3	91.4	91.5	91.4	95.9
**Top4**	90.1	82.0	89.9	90.1	90.0	95.0
**Top5**	90.8	85.9	90.9	90.8	90.8	96.3
**Top6**	89.4	83.6	89.5	89.4	89.4	95.7
**Top7**	90.1	82.0	89.9	90.1	90.0	95.6
**Top8**	90.1	83.8	90.1	90.1	90.1	96.1
**Top9**	91.5	86.1	91.5	91.5	91.5	96.5
**Top10**	91.5	86.1	91.5	91.5	91.5	96.5
**Top11**	92.2	88.2	92.3	92.2	92.2	97.3
**Top12**	92.9	90.3	93.1	92.9	93.0	97.5
**Top13**	92.2	88.2	92.3	92.2	92.2	97.2
**Top14**	92.2	88.2	92.3	92.2	92.2	97.3
**Top15**	93.6	90.5	93.7	93.6	93.6	97.3

### Characteristics of Identified Plasma Lipid Biomarkers

Because using top 15 lipid biomarkers together had the best predictive power in diagnosis of prostate cancer, the characteristics in this group of apparent lipid species were further analyzed.

**Figure 2:**
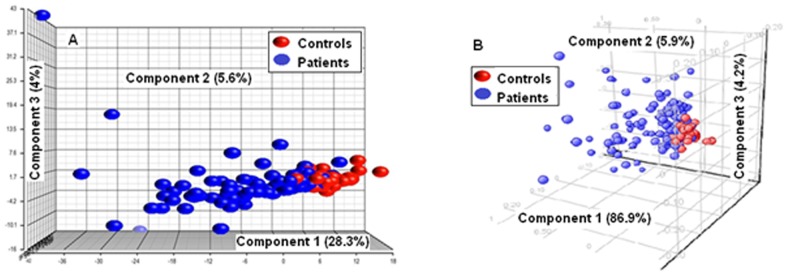
Comparison of Principal Component Analysis (PCA) with 390 and 15 selected plasma apparent lipid species. A: The first component in PCA cross all 390 detected plasma apparent lipid species accounts for 28.3% of the overall variance; B: The first component in PCA cross 15 selected plasma apparent lipid biomarkers accounts for 86.9% of the overall variance.

Principal components analysis (PCA) was performed to examine the ability to separate patient and control subjects with lipid profiles of all 390 and the selected 15 apparent lipid species. Performing PCA with lipid profiles of 390 apparent lipid species, patient and control subjects were visually separated along the first component in PCA, which accounted for 28.3% of the overall variance (Figure 2, A). However, when PCA was performed with lipid profiles of the selected 15 apparent lipid species, the first component, along which patient and control samples were separated, accounted for 86.9% of the overall variance in the data (Figure 2, B). This indicates that much more variations in these top 15 apparent lipid species are associated with the classification of patient or control. In addition, positions of control subjects were spatially closer than those of patient subjects when either set of data was plotted, suggesting that variances in mean plasma lipid concentration (here transformed to uncorrelated values of space distances) in the control group were much smaller than those in the patient group.

Hierarchical cluster analysis (HCA) is a statistical method for finding relatively homogeneous clusters of cases based on measured characteristics. We applied HCA combining dendrograms and a heatmap to cluster both entity (15 apparent lipid species) and condition (141 subjects) with panels of characteristics at the left side in Figure S1. The HCA analysis showed that 15 apparent lipid species were patterned into 4 clusters: DSM(16∶0), ether-linked phosphatidylcholines (ePCs), sphingomyelins (SMs) and LPCs. The 15 top ranking apparent lipid species had a tendency of gradually increasing mean plasma lipid concentration from top (lower concentration, in blue) to bottom (higher concentration, in red). To further analyze the data, the subjects in the heatmap were divided into two halves: the first half containing 70 subjects with lower plasma lipid concentration, and the second half containing 71 subjects with higher plasma lipid concentration. The analyzed results, as shown in Table 4, indicated that there is a dramatically high Odds Ratio in the presence of control and patient subjects between the first and second half (p<0.0001, OR = 70.0). Interestingly, patient subjects diagnosed by biopsies were significantly enriched in the second half as compared with those diagnosed by prostatectomies. When the subjects were stratified by age, race and Gleason grade of patients with prostate cancer, it showed that the second half (higher concentrations of individual apparent lipid species) had more black patients, especially black patients with old age, as compared with the first half. Cancer grade and overall age were not significantly different between two halves.

**Table 4:** Comparison of subject distributions and patient characteristics between upper and lower halves in the cluster of top 15 apparent lipid species.

	The first half	The second half	p value	OR	95% C.I.
**Total Controls (36)**	35	1			
**Total Patients (105)**	35	70	<0.0001	70	9.6–1430.5
**Patient Characteristics**					
**Ratio of Black to White**	0.40	0.84	0.09	0.48	0.18–1.20
**Ratio of biopsy to prostatectomy**	0.93	4.15	0.0007	4.75	1.69–13.59
**Ratio of high to low grade Pca**	0.67	0.50	0.65	0.75	0.28–1.99
**Patient age year-old (mean±SD)**	62.8±11.3	63±7.92	0.92		
**Ratio of Black∶White in young patients**	0.75	0.78	0.31	2.16	0.47–10.02
**Ratio of Black∶White in old patients**	0.27	0.93	0.07	0.92	0.06–1.30

It is unclear whether imbalanced compositions of race and age in this study cohort influenced the predictive power of the selected 15 apparent lipid species in diagnosis of prostate cancer. To clarify this issue, an “unknown prediction” method was applied. The results of unknown prediction are shown in Table 5. Using RandomForrest and 10-fold cross validation programs, satisfactory models in the training set were demonstrated by their high predictive powers in each group of subjects with a “known feature”, such as “white patient or control”, “young patient or control”, and in “random group 1” subjects. These results suggested that variability within any out of 10 iterations of subjects with a “known feature” did not affect the predictive powers of other iterations. Each group of subjects with a corresponding “unknown feature”, such as “black patient or control”, “old patient or control”, and in “random group 2” subjects in the test set, was cross validated by its corresponding established model in the training set. Similarities in high predictive powers in each set of paired groups indicated that the variability between the paired features (known and unknown), such as white vs. black, young vs. old and random group 1 subjects vs. random group 2 subjects, did not affect the selected 15 selected lipid biomarkers in differentiation of patients with prostate cancer from controls. Thus, imbalance of age and race between patient and control groups did not affect the ability of the selected 15 lipid biomarkers to differentiate patients from controls in this study cohort.

**Table 5:** Comparison of predictive values (%) of the top 15 plasma lipid biomarkers in diagnosis of prostate cancer in training set and testing set.

Predict	Group	Predictive values (%) in training set						Predictive values (%) in testing set					
Category		Sens.	Spec.	Prec.	Recall	F-m.	AUC	Sens.	Spec.	Prec.	Recall	F-m.	AUC
**White**	Patient	95.1	95.8	98.3	95.1	96.7	97.3	95.2	91.7	97.6	95.2	96.4	97.0
predict	Control	95.8	95.1	88.5	95.8	92.0	97.3	91.7	95.2	84.6	91.7	88.0	97.0
**black**	Weighted average	95.3	95.6	95.5	95.3	95.3	97.3	94.4	92.5	94.7	94.4	94.5	97.0
**Young**	Patient	96.5	86.4	94.8	96.5	95.7	97.6	93.8	78.6	93.8	93.8	93.8	96.9
**predict**	Control	86.4	96.5	90.5	86.4	88.4	97.6	78.6	93.7	78.6	78.6	78.6	96.9
**old**	Weighted average	93.7	89.2	93.6	93.7	93.6	97.6	90.3	82.0	90.3	90.3	90.3	96.9
**Group1**	Patient	92.9	86.4	95.6	92.9	94.2	95.6	94.3	85.7	94.3	94.3	94.3	94.1
**predict**	Control	86.4	92.9	79.2	86.4	82.6	95.6	85.7	94.3	85.7	85.7	85.7	94.1
**group 2**	Weighted average	91.3	87.9	91.7	91.3	91.4	95.6	91.8	88.2	91.8	91.8	91.8	94.1

***Sens. = Sensitivity, Spec. = Specificity, Prec. = Precision, F-m. = F-measure, AUC = Area under (ROC) curve.***

Among the 355 un-selected apparent lipid species, 43 (12.11%) of the lipid molecules contained saturated fatty acid chains only (no unsaturated fatty acid chains). While within the 35 selected apparent lipid species, 12 (34.3%) lipid molecules contain saturated fatty acid chains only. The difference in percentage of apparent lipid species containing saturated fatty acid only was highly significant between the selected 35 candidates and those un-selected apparent lipid species (p<0.001, diagnostic odds ratio was 3.79 with a 95% confidence interval of 1.64–8.67). These results suggested that the apparent lipid species with only saturated fatty acids chain only might play certain roles in pathogenesis of prostate cancer.

**Figure 3:**
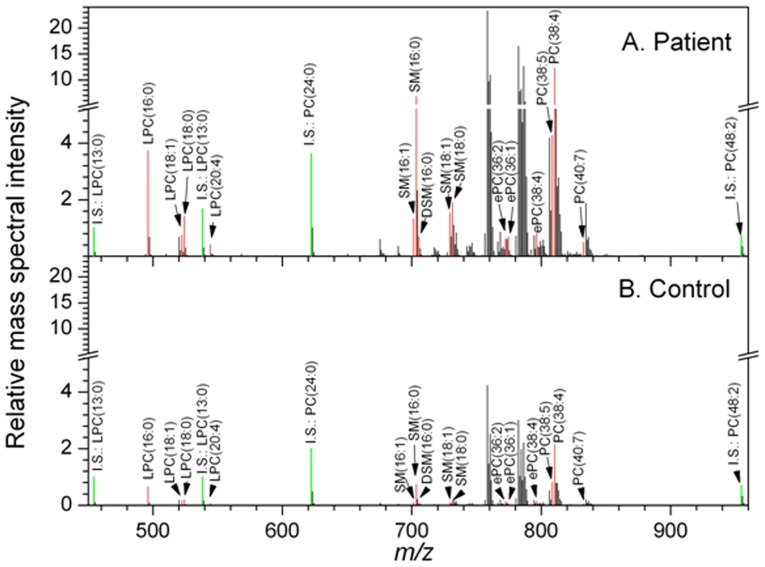
Mass spectra of phosphocholine-containing lipids (Pre-184 positive mode, including biomarker species. A: Spectra of 15 selected apparent lipid species in a representative patient with prostate cancer. B: Spectra of 15 selected apparent lipid species in a representative male control. Spectral intensities were normalized to that of internal standard LPC(13∶0). The intensities of phosphocholine-containing internal standards (I.S.) are indicated in green. The intensities of the identified biomarkers are shown in red. Internal standards and biomarkers (15 selected apparent lipid species) are labeled.

The mass spectra of top 15 apparent lipid species in representative patient and control samples are shown in Figure 3. Identified lipid species were exclusively phosphocholine-containing phospholipids, including 3 ePCs, 3 PCs, 4 LPCs and 5 SM/DSMs.

## Discussion

In clinical practice, PSA is the most commonly used biomarker for prostate cancer. However there is a substantial overlap in serum PSA level between patients with and without prostate cancer. No single PSA cutoff value can satisfactorily provide a simultaneously higher sensitivity and specificity in diagnosis of prostate cancer. Taking an example, one study showed that a PSA cutoff value of 1.1 ng/ml had a sensitivity of 83.4% with a specificity of 38.9%; that of 4.1 ng/ml had a sensitivity of 20.5% with a specificity of 93.8%; and the area under (ROC) curve was 68.2% [41]–[42]. Other newly reported biomarkers have been less systemically studied for their sensitivity, specificity and accuracy in diagnosis of prostate cancer. As compared to PSA, plasma lipid biomarkers identified in this study had much higher sensitivity, specificity and accuracy simultaneously in diagnosis of prostate cancer. It is especially true when multiple lipid markers were used together. For example, using the top 3 plasma lipid biomarkers together achieved a much higher predictive values in differentiation of prostate cancers from controls as compared to PSA. Of course, further investigations are needed to demonstrate if there are overestimations of sensitivity, specificity and accuracy due to the feature selections used in this pilot study.

Human plasma contains thousands of distinct lipid species. Although normal ranges of some lipid species have been determined in plasma [43]–[44], the majority have not. Recently, standard reference values for 500 plasma lipid molecules were produced by lipidomics on plasma samples pooled and blended from 100 healthy American people [23]. Many apparent lipid species in our study overlapped with those. Although some discrepancies were noted, comparison of mean plasma concentration of the apparent lipid species detected in both studies indicated that many values were extremely close, including several that we identified as individual lipid biomarkers for prostate cancer in our study. For example, LPC(18∶1), which was ranked as top 1 plasma lipid biomarker in our study, mean plasma concentration was 14.8 nmol/ml in the referenced study and 15.82 pmol/µl (i.e. nmol/ml) in the control group of our study. Similarly in LPC(20∶4), the second top plasma lipid biomarker in our study, mean plasma concentration was 5.37 nmol/ml in the referenced study, and 4.61 pmol/µl in the control group of our study. The reason for some discrepancies between two studies is unclear. In addition to the great variability in individual subject and lipid species, other possible reasons for these discrepancies could be the bias between different detecting centers, or biochemical interactions occurred in repeated freezing-thawing process in the referenced study, or the smaller sample size of the control group in our study. It is desirable to establish an international standard reference values with normal ranges for all plasma lipid species to meet with the increasing demand in lipid research and clinical practice.

Univariate analysis of one apparent lipid species is unlikely to be sufficient to discriminate prostate cancer patients from controls because of considerable variation of plasma lipid concentrations. A combination of multiple plasma lipid biomarkers with multivariate analysis and various classification algorithms was demonstrated herein to have enhanced predictive power in the diagnosis of prostate cancer in our study. A similar effect was reported by Landers et al [45], who found that combined 4 gene markers greatly increased sensitivity in discriminating prostate cancer from benign prostatic hyperplasia (BPH), and by Petricoin et al [46], who applied a genetic algorithm and clustering analysis to abstract discriminatory patterns of proteomics data from thousands of protein and peptide molecules to separate ovarian cancers from controls with a sensitivity of 100%, specificity of 95%, and positive predictive value of 94%. Measurement of a panel of a few plasma lipid molecules, for example 3 to 15 apparent lipid species may be feasible in routine clinical laboratories. The test requires very small amount of blood (3 µl of plasma), which can be obtained with minimally invasive procedures. The test results could be obtained within an hour. Together with all of these advantages of lipidomic technology, a new diagnostic model based on statistical and bioinformatic analysis with high predictive values could be used in diagnosis of prostate cancer in the near future. This study, once validated prospectively in our ongoing study, or confirmed by other researchers, potentially will have a revolutionary impact on diagnosis and study of the pathogenesis of prostate cancer, and other diseases.

Overall, patients with prostate cancer had increased plasma lipid concentrations in all lipid classes except PA, which was significantly lower in patients with prostate cancer. This imbalance in plasma concentrations among lipid classes could be caused by increased synthesis of other phospholipids in prostate cancer resulting in over-consumption of PA, because PA is the precursor for synthesis of many other phospholipids [47]. It is unclear why the plasma concentrations of individual lipid species in same lipid class were greatly varied: some increased and others decreased in the patients. One possible mechanism for this imbalance is that prostate cancer cells regulate the enzymes that control lipid metabolic pathways of not only synthesizing but also remodeling phospholipids. The expression level of one of these enzymes, lysophosphatidylcholine acyltransferase 1 (LPCAT1), a key enzyme in Lands' cycle remodeling pathway, correlated with the progression of prostate cancer [48]. We also found that nuclear translocation of soluble phospholipase A2 (sPLA2) was up-regulated in prostate cancer tissues as compared with benign prostatic tissues (unpublished), but it is not clear whether the nuclear translocation of sPLA2 is associated with remodeling of phospholipid. The fact that all identified top 15 apparent lipid species were species containing phosphocholine is very intriguing. This result might suggest up-regulation of phosphocholine metabolism in patient with prostate cancer, and is consistent with previous findings that high grade prostate cancer tissues had higher concentration of phosphocholine as compared with low grade prostate cancer [49].

This preliminary study has several limitations. First, more than 90% of plasma samples were obtained from prostate cancer patients with a Gleason score of 6 or 7, which made it impossible to correlate the identified plasma lipid biomarkers with the severity of the prostate cancer, as well as to the patients with metastasis or benign changes. Second, due to incomplete information on the patients' serum PSA level at the time of samples' collection, and to the lack of information on patients' outcomes, we were unable to compare the predictive power of the identified lipid biomarkers with that of PSA in the same study cohort. Therefore, at this point, the identified plasma apparent lipid species might serve as diagnostic biomarkers only, but not as prognostic and screening biomarkers. As well, the specificity of these identified lipid biomarkers has not been tested for other cancers and metabolic disorders. Even if there are limitations in the study, it is a pioneering work, exploring a new approach to seeking biomarkers for prostate cancer and other diseases.

## Supporting Information

Figure S1 Hierachical Cluster Analysis (HCA) based on 15 apparent lipid species and 141 subjectsand their clinical characteristics. Exel files.
(XLSX)

Table S1 Difference in plasma concentration of individual apparent lipid species between patient and control. Exel files.
(XLSX)

## References

[1] Ramírez ML, Nelson EC, Evans CP (2008) Beyond prostate-specific antigen: alternate serum markers. Prostate Cancer Prostatic Dis 11 3 216–29. 18227856 10.1038/pcan.2008.2

[2] Draisma G, Etzioni R, Tsodikov A, Mariotto A, Wever E, et al (2009) Lead time and overdiagnosis in prostate-specific antigen screening: importance of methods and context. J Natl Cancer Inst 101 6 374–83. 19276453 10.1093/jnci/djp001 PMC2720697

[3] Etzioni R, Penson DF, Legler JM, di Tommaso D, Boer R, et al (2002) Over diagnosis due to prostate-specific antigen screening: lessons from U.S. prostate cancer incidence trends. Natl Cancer Inst 94 13 981–90. 10.1093/jnci/94.13.981 12096083

[4] Hamilton RJ, Platz EA, Freedland SJ (2009) Re: Prostate-specific antigen: a misused and maligned prostate cancer biomarker. J Natl Cancer Inst 101 8 611–2. 19351913 10.1093/jnci/djp043

[5] Elgamal AA, Holmes EH, Su SL, Tino WT, Simmons SJ, et al (2000) Prostate-specific membrane antigen (PSMA): current benefits and future value. Semin Surg Oncol 18 1 10–6. 10617892 10.1002/(sici)1098-2388(200001/02)18:1<10::aid-ssu3>3.0.co;2-v

[6] Kirby RS, Fitzpatrick JM, Irani J (2009) Prostate cancer diagnosis in the new millennium: strengths and weaknesses of prostate-specific antigen and the discovery and clinical evaluation of prostate cancer gene 3 (PCA3). BJU Int 103 4 441–5. 19154510 10.1111/j.1464-410X.2008.08280.x

[7] Rogers CG, Yan G, Zha S, Gonzalgo ML, Isaacs WB, et al (2004) Prostate cancer detection on urinalysis for alpha methylacyl coenzyme a racemase protein. J Urol 172 4 Pt 1 1501–3. 15371879 10.1097/01.ju.0000137659.53129.14

[8] Crowe FL, Allen NE, Appleby PN (2008) Fatty acid composition of plasma phospholipids and risk of prostate cancer in a case-control analysis nested within the European Prospective Investigation into Cancer and Nutrition. Am J Clin Nutr 88: 1353–63. 18996872 10.3945/ajcn.2008.26369

[9] Freeman VL, Flanigan RC, Meydani M (2007) Prostatic fatty acids and cancer recurrence after radical prostatectomy for early-stage prostate cancer. Cancer Causes Control 18 2 211–8. 17216324 10.1007/s10552-006-0095-6

[10] Teichert F, Verschoyle RD, Greaves P, Edwards RE, Teahan O, et al (2008) Metabolic profiling of transgenic adenocarcinoma of mouse prostate (TRAMP) tissue by 1H-NMR analysis: evidence for unusual phospholipid metabolism. Prostate 68 10 1035–47. 18459103 10.1002/pros.20761

[11] Graff JR, Konicek BW, McNulty AM, Wang Z, Houck K, et al (2000) Increased AKT activity contributes to prostate cancer progression by dramatically accelerating prostate tumor growth and diminishing p27Kip1 expression. J Biol Chem 275: 24500–245056. 10827191 10.1074/jbc.M003145200

[12] Xie Y, Gibbs TC, Mukhin YV, Meier KE (2002) Role for 18∶1 lysophosphatidic acid as an autocrine mediator in prostate cancer cells. J Biol Chem 277 36 32516–26. 12084719 10.1074/jbc.M203864200

[13] Zeng Y, Kakehi Y, Nouh MA, Tsunemori H, Sugimoto M, et al (2009) Gene expression profiles of lysophosphatidic acid-related molecules in the prostate: relevance to prostate cancer and benign hyperplasia. Prostate 69 3 283–92. 19025891 10.1002/pros.20879

[14] Kurhanewicz J, Thomas A, Jajodia P, Weiner MW, James TL, et al (1991) 31P spectroscopy of the human prostate gland in vivo using a transrectal probe. Magn Reson Med 22 2 404–13. 1725918 10.1002/mrm.1910220248

[15] Swanson MG, Keshari KR, Tabatabai ZL, Simko JP, Shinohara K, et al (2008) Quantification of choline- and ethanolamine-containing metabolites in human prostate tissues using 1H HR-MAS total correlation spectroscopy. Magn Reson Med 60 1 33–40. 18581409 10.1002/mrm.21647 PMC2643975

[16] Pchejetski D, Golzio M, Bonhoure E, Calvet C, Doumerc N, et al (2005) Sphingosine kinase-1 as a chemotherapy sensor in prostate adenocarcinoma cell and mouse models. Cancer Res 65 24 11667–75. 16357178 10.1158/0008-5472.CAN-05-2702

[17] Gibbs TC, Rubio MV, Zhang Z, Xie Y, Kipp KR, et al (2009) Signal transduction responses to lysophosphatidic acid and sphingosine 1-phosphate in human prostate cancer cells. Prostate 69 14 1493–506. 19536794 10.1002/pros.20994

[18] Bravi F, Bosetti C, Scotti L, Talamini R, Montella M, et al (2007) Food groups and renal cell carcinoma: a case-control study from Italy. Int J Cancer 120 3 681–5. 17058282 10.1002/ijc.22225

[19] Zhuang L, Kim J, Adam RM, Solomon KR, Freeman MR (2005) Cholesterol targeting alters lipid raft composition and cell survival in prostate cancer cells and xenografts. J Clin Invest 115 4 959–68. 15776112 10.1172/JCI200519935 PMC1064980

[20] Simons K, Ikonen E (1997) Functional rafts in cell membranes. Nature 387 6633 569–72. 9177342 10.1038/42408

[21] Pflug BR, Pecher SM, Brink AW, Nelson JB, Foster BA (2003) Increased fatty acid synthase expression and activity during progression of prostate cancer in the TRAMP model. Prostate 57 3 245–54. 14518031 10.1002/pros.10297

[22] Han X, Gross RW (2003) Global analyses of cellular lipidomes directly from crude extracts of biological samples by ESI mass spectrometry: a bridge to lipidomics. J Lipid Res 44 6 1071–9. 12671038 10.1194/jlr.R300004-JLR200

[23] Quehenberger O, Armando AM, Brown AH, Milne SB, Myers DS, et al (2010) Lipidomics reveals a remarkable diversity of lipids in human plasma. J Lipid Res 51 11 3299–305. 20671299 10.1194/jlr.M009449 PMC2952570

[24] Kolak M, Westerbacka J, Velagapudi VR, Wågsäter D, Yetukuri L, et al (2007) Adipose tissue inflammation and increased ceramide content characterize subjects with high liver fat content independent of obesity. Diabetes 56 8 1960–8. 17620421 10.2337/db07-0111

[25] Pietiläinen KH, Sysi-Aho M, Rissanen A, Seppänen-Laakso T, Yki-Järvinen H, et al (2007) Acquired obesity is associated with changes in the serum lipidomic profile independent of genetic effects–a monozygotic twin study. PLoS One 2 2 e218. 17299598 10.1371/journal.pone.0000218 PMC1789242

[26] Ekroos K, Jänis M, Tarasov K, Hurme R, Laaksonen R (2010) Lipidomics: A Tool for Studies of Atherosclerosis. Curr Atheroscler Rep 2010 Apr 28. 10.1007/s11883-010-0110-y PMC2878593 20425241

[27] Lankinen M, Schwab U, Erkkilä A, Seppänen-Laakso T, Hannila ML, et al (2009) Fatty fish intake decreases lipids related to inflammation and insulin signaling–a lipidomics approach. PLoS One 4 4 e5258. 19390588 10.1371/journal.pone.0005258 PMC2669180

[28] de Mello VD, Lankinen M, Schwab U, Kolehmainen M, Lehto S, et al (2009) Link between plasma ceramides, inflammation and insulin resistance: association with serum IL-6 concentration in patients with coronary heart disease. Diabetologia 52 12 2612–5. 19669729 10.1007/s00125-009-1482-9

[29] Graessler J, Schwudke D, Schwarz PE, Herzog R, Shevchenko A, et al (2009) Top-down lipidomics reveals ether lipid deficiency in blood plasma of hypertensive patients. PLoS One 4 7 e6261. 19603071 10.1371/journal.pone.0006261 PMC2705678

[30] Han X, Yang J, Yang K, Zhao Z, Abendschein DR, et al (2007) Alterations in myocardial cardiolipin content and composition occur at the very earliest stages of diabetes: a shotgun lipidomics study. Biochemistry 46 21 6417–28. 17487985 10.1021/bi7004015 PMC2139909

[31] Ollero M, Astarita G, Guerrera IC, Sermet-Gaudelus I, Trudel S, et al (2011) Plasma lipidomics reveals potential prognostic signatures within a cohort of cystic fibrosis patients. J Lipid Res 52 5 1011–22. 21335323 10.1194/jlr.P013722 PMC3073467

[32] Görke R, Meyer-Bäse A, Wagner D, He H, Emmett MR, et al (2010) Determining and interpreting correlations in lipidomic networks found in glioblastoma cells. BMC Syst Biol 4: 126. 20819237 10.1186/1752-0509-4-126 PMC2944140

[33] He H, Conrad CA, Nilsson CL, Ji Y, Schaub TM, et al (2007) Method for lipidomic analysis: p53 expression modulation of sulfatide, ganglioside, and phospholipid composition of U87 MG glioblastoma cells. Anal Chem 79 22 8423–30. 17929901 10.1021/ac071413m PMC4851466

[34] Balogh G, Péter M, Liebisch G, Horváth I, Török Z, et al (2010) Lipidomics reveals membrane lipid remodelling and release of potential lipid mediators during early stress responses in a murine melanoma cell line. Biochim Biophys Acta 1801 9 1036–47. 20430110 10.1016/j.bbalip.2010.04.011

[35] Min HK, Lim S, Chung BC, Moon MH (2011) Shotgun lipidomics for candidate biomarkers of urinary phospholipids in prostate cancer. Anal Bioanal Chem 399 2 823–30. 20953865 10.1007/s00216-010-4290-7

[36] Devaiah S, Roth M, Baughman E, Li M, Tamura P, et al (2006) Quantitative profiling of polar glycerolipid species from organs of wild-type Arabidopsis and a phospholipase Dal knockout mutant. Phytochemistry 67: 1907–1924. 16843506 10.1016/j.phytochem.2006.06.005

[37] Bartz R, Li WH, Venables B, Zehmer J, Welti R, et al (2007) Lipidomics reveals adiposomes store ether lipids and mediate phospholipid traffic. J Lipid Res 48: 837–847. 17210984 10.1194/jlr.M600413-JLR200

[38] Welti R, Li W, Li M, Sang Y, Biesiada H, et al (2002) Profiling membrane lipids in plant stress responses: Role of phospholipase D{alpha} in freezing-induced lipid changes in Arabidopsis. J. Biol. Chem 277: 31994–32002. 10.1074/jbc.M205375200 12077151

[39] Brügger B, Erben G, Sandhoff R, Wieland FT, Lehmann WD (1997) Quantitative analysis of biological membrane lipids at the low picomole level by nano-electrospray ionization tandem mass spectrometry. Proc Natl Acad Sci U S A 94 6 2339–44. 9122196 10.1073/pnas.94.6.2339 PMC20089

[40] Liebisch G, Lieser B, Rathenberg J, Drobnik W, Schmitz G (2004) High-throughput quantification of phosphatidylcholine and sphingomyelin by electrospray ionization tandem mass spectrometry coupled with isotope correction algorithm. Biochim Biophys Acta 1686 1–2 108–17. 15522827 10.1016/j.bbalip.2004.09.003

[41] Thompson IM, Ankerst DP, Chi C, Lucia MS, Goodman PJ, et al (2005) Operating characteristics of prostate-specific antigen in men with an initial PSA level of 3.0 ng/ml or lower. JAMA 294 1 66–70 PMID: 15998892. 15998892 10.1001/jama.294.1.66

[42] Thompson IM, Ankerst DP (2007) Prostate-specific antigen in the early detection of prostate cancer. CMAJ 176 13 1853–8. 17576986 10.1503/cmaj.060955 PMC1891131

[43] Ding J, Sorensen CM, Jaitly N, Jiang H, Orton DJ, et al (2008) Application of the accurate mass and time tag approach in studies of the human blood lipidome. J Chromatogr B Analyt Technol Biomed Life Sci 871 2 243–52. 10.1016/j.jchromb.2008.04.040 PMC2598838 18502191

[44] Graessler J, Schwudke D, Schwarz PE, Herzog R, Shevchenko A, et al (2009) Top-down lipidomics reveals ether lipid deficiency in blood plasma of hypertensive patients. PLoS One 4 7 e6261. 19603071 10.1371/journal.pone.0006261 PMC2705678

[45] Landers KA, Burger MJ, Tebay MA, Purdie DM, Scells B, et al (2005) Use of multiple biomarkers for a molecular diagnosis of prostate cancer. Int J Cancer 114 6 950–6. 15609297 10.1002/ijc.20760

[46] Petricoin EF, Ardekani AM, Hitt BA, Levine PJ, Fusaro VA, et al (2002) Use of proteomic patterns in serum to identify ovarian cancer. Lancet 359 9306 572–7. 11867112 10.1016/S0140-6736(02)07746-2

[47] Athenstaedt K, Daum G (1999) Phosphatidic acid, a key intermediate in lipid metabolism. SourceInstitut für Biochemie, Technische Universität, Graz, Austria. Eur J Biochem 266 1 1–16. 10542045 10.1046/j.1432-1327.1999.00822.x

[48] Zhou X, Lawrence TJ, He Z, Pound CR, Mao J, et al (2011) The expression level of lysophosphatidylcholine acyltransferase 1 (LPCAT1) correlates to the progression of prostate cancer. Exp Mol Pathol 92 1 105–10. 22101258 10.1016/j.yexmp.2011.11.001 PMC3294031

[49] Keshari KR, Tsachres H, Iman R, Delos Santos L, Tabatabai ZL, et al (2011) Correlation of phospholipid metabolites with prostate cancer pathologic grade, proliferative status and surgical stage - impact of tissue environment. NMR Biomed 24 6 691–9 doi:10.1002/nbm.1738. 21793074 10.1002/nbm.1738 PMC3653775

